# A modified belief entropy in Dempster-Shafer framework

**DOI:** 10.1371/journal.pone.0176832

**Published:** 2017-05-08

**Authors:** Deyun Zhou, Yongchuan Tang, Wen Jiang

**Affiliations:** School of Electronics and Information, Northwestern Polytechnical University, Xi’an, Shaanxi, China; Nankai University, CHINA

## Abstract

How to quantify the uncertain information in the framework of Dempster-Shafer evidence theory is still an open issue. Quite a few uncertainty measures have been proposed in Dempster-Shafer framework, however, the existing studies mainly focus on the mass function itself, the available information represented by the scale of the frame of discernment (FOD) in the body of evidence is ignored. Without taking full advantage of the information in the body of evidence, the existing methods are somehow not that efficient. In this paper, a modified belief entropy is proposed by considering the scale of FOD and the relative scale of a focal element with respect to FOD. Inspired by Deng entropy, the new belief entropy is consistent with Shannon entropy in the sense of probability consistency. What’s more, with less information loss, the new measure can overcome the shortage of some other uncertainty measures. A few numerical examples and a case study are presented to show the efficiency and superiority of the proposed method.

## 1 Introduction

Dempster-Shafer evidence theory [1, 2] is effective in modeling and processing uncertain information of intelligent systems. It has been extensively studied in many fields such as pattern recognition [3–8], fault diagnosis [9–12], multiple attribute decision making [13–15], risk analysis [16–20], controller design [21, 22] and so on [23–25]. However, some open issues in Dempster–Shafer evidence theory are still needed for further study. Firstly, highly conflicting evidence may lead to counterintuitive results, conflict management among different information sources should be addressed cautiously [26–29]. Secondly, the dependence among different evidence should be taken into consideration before applying combination rule [30–32]. Thirdly, a widely applicable method of generating basic probability assignment (BPA) should be developed to model uncertain information [33–35]. Finally, the incompleteness of the frame of discernment (FOD) should be taken into consideration in an open world [29, 36–38]. These open issues are often related to uncertainty modeling. One way to manage the uncertainty is to quantify the uncertainty before further information processing.

Uncertainty often comes from several types of uncertain and incomplete information, including ignorance, vagueness and so on [39]. Uncertainty and ignorance are difficult categories to deal with [40]. Although ignorance increases the uncertain degree of uncertain information in an open world, with a proper uncertainty measure, one can manage or even decrease the uncertain degree of uncertain information. Since uncertainty measure is a hot topic in information processing [41–44], many theories have been developed for uncertainty modeling, such as Shannon entropy [45], possibility theory [46], fuzzy sets [47], Dempster–Shafer evidence theory [1, 2] and rough sets [48]. Some extended theories and hybrid methods are also presented for uncertainty measure, e.g. Hohle’s confusion measure [49], Yager’s dissonance measure [50], the weighted Hartley entropy [51], Klir & Ramer’s discord measure [52] and Klir & Parviz’s strife measure [53], Deng entropy [54], generalized evidence theory [29], D numbers [55] and so on [56–61]. Among these methods, Shannon entropy is a well-known theory for uncertainty measure in the probabilistic framework. For example, as a generalization of Shannon entropy, network entropy is an effective measurement for testing the complexity of networks [62–65]. But Shannon entropy can’t be used directly in the framework of Dempster–Shafer evidence theory, because a mass function in evidence theory is a generalized probability assigned on the power set of FOD. To address this issue, some modified methods based on Shannon entropy are proposed [49–53], of which some have been successfully applied in real applications [66, 67]. However, these methods are somehow not that effective in some cases [54, 56].

Recently, in Dempster–Shafer framework, a new uncertainty measure named Deng entropy is proposed. Deng entropy can measure the uncertain degree more efficiently than some other uncertainty measures in some cases [54]. Although Deng entropy has been successfully applied in some real applications [9–12, 68], it doesn’t take into consideration of the scale of the FOD, which means a loss of available information while doing information processing. The information loss will lead to fail in uncertainty measure in some cases. In order to overcome this shortage of Deng entropy, a modified belief entropy based on Deng entropy is proposed in this paper. The proposed belief entropy can improve the performance of Deng entropy by considering the scale of the FOD and the relative scale of a focal element with respect to FOD. What’s more, the proposed method remains all the merits of Deng entropy thus it can degenerate to Shannon entropy in the sense of the probability consistency.

The rest of this paper is organized as follows. In Section 2, the preliminaries on Dempster–Shafer evidence theory, Shannon entropy, Deng entropy and some uncertainty measures in Dempster–Shafer framework are briefly introduced. In Section 3, the new belief entropy is presented. In Section 4, some numerical examples are presented, as well as a comparative study between the new belief entropy and some other uncertainty measures. In Section 5, a case study is presented to show the effectiveness and the potential application prospect of the new measure. The conclusions and ongoing work are given in Section 6.

## 2 Preliminaries

Some preliminaries are briefly introduced in this section, including Dempster-Shafer evidence theory, Shannon entropy, Deng entropy and some other typical uncertainty measures in Dempster-Shafer framework.

### 2.1 Dempster-Shafer evidence theory

Let Ω = {*θ*_1_, *θ*_2_, …, *θ*_*i*_, …, *θ*_*N*_} be be a finite nonempty set of mutually exclusive and exhaustive events, Ω is called the *frame of discernment* (FOD). The power set of Ω, denoted as 2^Ω^, is composed of 2^*N*^ elements denoted as follows:

$$\begin{array}{c} 2^{\Omega }=\left\{\emptyset ,\left\{\theta_{1}\right\},\left\{\theta_{2}\right\},\ldots ,\left\{\theta_{N}\right\},\left\{\theta_{1},\theta_{2}\right\},\ldots ,\left\{\theta_{1},\theta_{2},\ldots ,\theta_{i}\right\},\ldots ,\Omega \right\}. \end{array}$$ (1)

A *mass function* *m* is defined as a mapping from the power set 2^Ω^ to the interval [0, 1], which satisfies the following conditions [1, 2]:

$$\begin{array}{c} m\left(\emptyset \right)=0,\;\sum_{A\in \Omega }m(A)=1. \end{array}$$ (2)

If *m*(*A*) > 0, then *A* is called a *focal element*, the mass function *m*(*A*) represents how strongly the evidence supports the proposition *A*.

A *body of evidence* (BOE), also known as a *basic probability assignment* (BPA) or *basic belief assignment* (BBA), is represented by the focal sets and their associated mass value:

$$\begin{array}{c} \left(ℜ,m\right)=\left\{\left\langle A,m(A)\right\rangle :A\in 2^{\Omega },m\left(A\right)>0\right\}. \end{array}$$ (3)

where ℜ is a subset of the power set 2^Ω^, each *A* ∈ ℜ has an associated nonzero mass value *m*(*A*).

A BPA *m* can also be represented by its associate belief function *Bel* and plausibility function *Pl* respectively, defined as follows:

$$\begin{array}{c} Bel\left(A\right)=\sum_{\phi \neq B\subseteq A}m(B)\;and\;Pl\left(A\right)=\sum_{B\cap A\neq \phi }m(B). \end{array}$$ (4)

In Dempster-Shafer evidence theory, two independent mass functions, denoted as *m*_1_ and *m*_2_, can be combined with Dempster’s rule of combination defined as [1, 2]:

$$\begin{array}{c} m\left(A\right)=\left(m_{1}⊕m_{2}\right)\left(A\right)\;=\frac{1}{1-k}\sum_{B\cap C=A}m_{1}\left(B\right)m_{2}\left(C\right), \end{array}$$ (5)

where *k* is a normalization constant representing the *degree of conflict* between *m*_1_ and *m*_2_, *k* is defined as [1, 2]:

$$\begin{array}{c} k=\sum_{B\cap C=\emptyset }m_{1}\left(B\right)m_{2}\left(C\right). \end{array}$$ (6)

### 2.2 Shannon entropy

As an uncertainty measure of information volume in a system or process, Shannon entropy plays a central role in information theory. Shannon entropy indicates that the information volume of each piece of information is directly connected to its uncertain degree.

Shannon entropy, as the information entropy, is defined as follows [45]:

$$\begin{array}{c} H=-\sum_{i=1}^{N}p_{i}\log_{b}p_{i}, \end{array}$$ (7)

where *N* is the number of basic states, *p*_*i*_ is the probability of state *i*, *p*_*i*_ satisfies $\sum_{i=1}^{N}p_{i}=1$. If the unit of information is bit, then *b* = 2.

### 2.3 Deng entropy

Deng entropy is a generalization of Shannon entropy in Dempster–Shafer framework [54]. If the information is modelled in the framework of a probability theory, Deng entropy can be degenerated to Shannon entropy. Deng entropy, denoted as *E*_*d*_, is defined as follows [54]:

$$\begin{array}{c} E_{d}\left(m\right)=-\sum_{A\subseteq X}m\left(A\right)\log_{2}\frac{m(A)}{2^{\left|A\right|}-1}, \end{array}$$ (8)

where |*A*| denotes the cardinality of the proposition *A*, *X* is the FOD. If and only if the mass value is assigned to single elements, Deng entropy can be degenerated to Shannon entropy, in this case, the form of Deng entropy is as follows:

$$\begin{array}{c} E_{d}\left(m\right)=-\sum_{A\subseteq X}m\left(A\right)\log_{2}\frac{m(A)}{2^{\left|A\right|}-1}=-\sum_{A\subseteq X}m\left(A\right)\log_{2}m\left(A\right). \end{array}$$ (9)

For more details about Deng entropy, please refer to [54].

### 2.4 Uncertainty measures in Dempster-Shafer framework

Assume that *X* is the FOD, *A* and *B* are focal elements of the mass function, and |*A*| denotes the cardinality of *A*. Then, the definitions of some typical uncertainty measures in Dempster-Shafer framework are briefly introduced as follows.

#### 2.4.1 Hohle’s confusion measure

Hohle’s confusion measure, denoted as *C*_*H*_, is defined as follows [49]:

$$\begin{array}{c} C_{H}\left(m\right)=-\sum_{A\subseteq X}m\left(A\right)\log_{2}Bel\left(A\right). \end{array}$$ (10)

#### 2.4.2 Yager’s dissonance measure

Yager’s dissonance measure, denoted as *E*_*Y*_, is defined as follows [50]:

$$\begin{array}{c} E_{Y}\left(m\right)=-\sum_{A\subseteq X}m\left(A\right)\log_{2}Pl\left(A\right). \end{array}$$ (11)

#### 2.4.3 Dubois & Prade’s weighted Hartley entropy

Dubois & Prade’s weighted Hartley entropy, denoted as *E*_*DP*_, is defined as follows [51]:

$$\begin{array}{c} E_{DP}\left(m\right)=\sum_{A\subseteq X}m\left(A\right)\log_{2}\left|A\right|. \end{array}$$ (12)

#### 2.4.4 Klir & Ramer’s discord measure

Klir & Ramer’s discord measure, denoted as *D*_*KR*_, is defined as follows [52]:

$$\begin{array}{c} D_{KR}\left(m\right)=-\sum_{A\subseteq X}m\left(A\right)\log_{2}\sum_{B\subseteq X}m\left(B\right)\frac{\left|A\cap B\right|}{\left|B\right|}. \end{array}$$ (13)

#### 2.4.5 Klir & Parviz’s strife measure

Klir & Parviz’s strife measure, denoted as *S*_*KP*_, is defined as follows [53]:

$$\begin{array}{c} S_{KP}\left(m\right)=-\sum_{A\subseteq X}m\left(A\right)\log_{2}\sum_{B\subseteq X}m\left(B\right)\frac{\left|A\cap B\right|}{\left|A\right|}. \end{array}$$ (14)

#### 2.4.6 George & Pal’s conflict measure

The total conflict measure proposed by George & Pal, denoted as *TC*_*GP*_, is defined as follows [56]:

$$\begin{array}{c} TC_{GP}\left(m\right)=\sum_{A\subseteq X}m\left(A\right)\sum_{B\subseteq X}m\left(B\right)\left(1-\frac{\left|A\cap B\right|}{\left|A\cup B\right|}\right). \end{array}$$ (15)

## 3 The proposed belief entropy

### 3.1 Problem description

In the framework of Dempster-Shafer evidence theory, the uncertain information is modeled not only by mass functions, the FOD is also the source of uncertainty, e.g. the number of elements in the FOD. However, Dubois & Prade’s weighted Hartley entropy and Deng entropy measure the uncertain degree of BOEs by only taking into consideration of the mass function and the cardinality of a proposition, the scale of FOD is totally ignored. Thus these methods can’t effectively measure the difference of uncertain degree with similar basic probability assignment on different FODs. A simple example of the limitation of Deng entropy and weighted Hartley entropy is shown in Example 3.1.

**Example 3.1.** Consider a target identification problem, assume that two reliable sensors report the detection results independently. The results are represented by BOEs shown as follows:

$$\begin{array}{ccc} m_{1}:m_{1}\left(\left\{a,b\right\}\right) & = & 0.4,m_{1}\left(\left\{c,d\right\}\right)=0.6.\\ m_{2}:m_{2}\left(\left\{a,c\right\}\right) & = & 0.4,m_{2}\left(\left\{b,c\right\}\right)=0.6. \end{array}$$

Recall the Eq (8) of Deng entropy, the uncertainty measure of *m*_1_ and *m*_2_ are shown as follows:

$$\begin{array}{c} E_{d}\left(m_{1}\right)=-\sum_{A\subseteq X}m_{1}\left(A\right)\log_{2}\frac{m_{1}\left(A\right)}{2^{\left|A\right|}-1}=-0.4\log_{2}\frac{0.4}{2^{2}-1}-0.6\log_{2}\frac{0.6}{2^{2}-1}=2.5559, \end{array}$$ (16)

$$\begin{array}{c} E_{d}\left(m_{2}\right)=-\sum_{A\subseteq X}m_{2}\left(A\right)\log_{2}\frac{m_{2}\left(A\right)}{2^{\left|A\right|}-1}=-0.4\log_{2}\frac{0.4}{2^{2}-1}-0.6\log_{2}\frac{0.6}{2^{2}-1}=2.5559. \end{array}$$ (17)

Recall the Eq (12) of Dubois & Prade’s weighted Hartley entropy, the uncertainty measure of *m*_1_ and *m*_2_ are shown as follows:

$$\begin{array}{c} E_{DP}\left(m_{1}\right)=\sum_{A\subseteq X}m_{1}\left(A\right)\log_{2}\left|A\right|=0.4\log_{2}2+0.6\log_{2}2=1, \end{array}$$ (18)

$$\begin{array}{c} E_{DP}\left(m_{2}\right)=\sum_{A\subseteq X}m_{2}\left(A\right)\log_{2}\left|A\right|=0.4\log_{2}2+0.6\log_{2}2=1. \end{array}$$ (19)

The results calculated by Deng entropy and the weighted Hartley entropy are counterintuitive. The two BOEs have the same mass value assignment, but the FOD of the first BOE consists of four targets denoted as *a*, *b*, *c* and *d*, while the second BOE has only three possible targets denoted as *a*, *b* and *c*. Intuitively, it is expected that the second BOE has less uncertainty than the first one. In other words, the uncertain degree of *m*_1_ should be bigger than that of *m*_2_. Both Deng entropy and weighted Hartley entropy fail to quantify the difference of uncertain degree among these two BOEs. To address this issue, a modified belief entropy based on Deng entropy is proposed.

### 3.2 The new belief entropy

In the framework of Dempster-Shafer evidence theory, the new belief entropy based on Deng entropy is shown as follows:

$$\begin{array}{c} E_{Md}\left(m\right)=-\sum_{A\subseteq X}m\left(A\right)\log_{2}\left(\frac{m(A)}{2^{\left|A\right|}-1}e^{\frac{|A|-1}{\left|X\right|}}\right), \end{array}$$ (20)

where |*A*| denotes the cardinality of the focal element *A*, |*X*| denotes the cardinality of *X* which represents the number of element in FOD. Compared with Deng entropy, the new belief entropy addresses more information in BOE, including the scale of FOD, denoted as |*X*|, and the relative scale of a focal element with respect to FOD, denoted as ((|*A*| − 1)/|*X*|).

The exponential factor $e^{\frac{|A|-1}{\left|X\right|}}$ in the new belief entropy represents the uncertain information in a BOE that has been ignored by Deng entropy and some other uncertainty measures such as the confusion measure, the dissonance measure, the weighted Hartley entropy, the discord measure and the strife measure. More importantly, by involving the scale of FOD in the proposed belief entropy, the new uncertainty measure now can effectively quantify the difference among different BOEs even if the same mass value is assigned on different FODs. In addition, the new information exponential factor doesn’t affect the merit of Deng entropy, which will be discussed in detail in the ensuing part of this paper.

With the new belief entropy, recall Example 3.1, the new belief entropy for these two BOEs is calculated as follows:

$$\begin{array}{ccc} E_{Md}\left(m_{1}\right) & = & -\sum_{A\subseteq X}m_{1}\left(A\right)\log_{2}\left(\frac{m_{1}\left(A\right)}{2^{\left|A\right|}-1}e^{\frac{|A|-1}{\left|X\right|}}\right)\\ & = & -0.4\log_{2}\left(\frac{0.4}{2^{2}-1}e^{\frac{2-1}{4}}\right)-0.6\log_{2}\left(\frac{0.6}{2^{2}-1}e^{\frac{2-1}{4}}\right)=2.1952, \end{array}$$ (21)

$$\begin{array}{ccc} E_{Md}\left(m_{2}\right) & = & -\sum_{A\subseteq X}m_{2}\left(A\right)\log_{2}\left(\frac{m_{2}\left(A\right)}{2^{\left|A\right|}-1}e^{\frac{|A|-1}{\left|X\right|}}\right)\\ & = & -0.4\log_{2}\left(\frac{0.4}{2^{2}-1}e^{\frac{2-1}{3}}\right)-0.6\log_{2}\left(\frac{0.6}{2^{2}-1}e^{\frac{2-1}{3}}\right)=2.0750. \end{array}$$ (22)

The comparison results of different uncertainty measures for Example 3.1 are shown in Table 1. It can be concluded that both Dubois & Prade’s weighted Hartley entropy and Deng entropy can’t measure the difference of uncertain degree between these two BOEs, while the new belief entropy can effectively measure the different uncertain degree by taking into consideration of more available information in the BOE. In addition, according to Table 1, the first BOE *m*_1_ has a higher uncertain degree with the new belief entropy, this is reasonable because the FOD of *m*_1_ consists of four candidate targets which means a larger information volume than the second BOE *m*_2_. The efficiency of the new belief entropy is not available in the weighted Hartley entropy and Deng entropy.

**Table 1:** Uncertainty measure of Example 3.1 with different methods.

BOEs	Weight Hartley entropy [51]	Deng entropy [54]	The modified belief entropy
*m*_1_	1	2.5559	2.1952
*m*_2_	1	2.5559	2.0750

### 3.3 Property of the new belief entropy

Some properties of the new belief entropy are presented in this section, including the range of the new measure and its compatibility with Shannon entropy.

**Property 1.** Mathematically, the value range of the new belief entropy is (0, +∞).

**Proof.** According to Dempster-Shafer evidence theory, a focal element *A* consists at least one element and the superior limit of its element number is the scale of FOD, while a FOD Ω (the *X* in Eq (20)) consists at least one element and there is no superior limit, thus the range of |*A*| and |*X*| are the same, denoted as [1, +∞). The range of a mass function *m*(*A*) is (0, 1].

Recall Eq (20), where |*A*| ∈ [1, +∞), |*X*| ∈ [1, +∞), *m*(*A*) ∈ (0, 1]. Thus the range of the new belief entropy can be denoted as *E*_*Md*_(*m*) ∈ (0, +∞).

**Property 2.** The new belief entropy can degenerate to the Shannon entropy when the mass function is Bayesian.

**Proof**: Recall Eq (20), if the mass function *m*(*A*) is Bayesian, then the mass value (BPA) is assigned only on single element subset, then |*A*|≡1. In this case, the new belief entropy can degenerate to the following equation:

$$\begin{array}{c} E_{Md}\left(m\right)=-\sum_{A\subseteq X}m\left(A\right)\log_{2}\left(\frac{m(A)}{2^{\left|A\right|}-1}e^{\frac{|A|-1}{\left|X\right|}}\right)=-\sum_{A\subseteq X}m\left(A\right)\log_{2}\left(\frac{m(A)}{2^{1}-1}e^{\frac{1-1}{\left|X\right|}}\right)=-\sum_{A\subseteq X}m\left(A\right)\log_{2}m\left(A\right). \end{array}$$ (23)

Eq (23) is in consistent with Eqs (9) and (7) when the mass function is Bayesian, because a mass function *m*(*A*) can degenerate to a Bayesian probability *p*_*i*_ in the sense of the probability consistency.

## 4 Numerical example and discussion

In order to show the rationality and merit of the proposed belief entropy, some numerical examples are presented in this section. In Section 4.1, the compatibility of the new belief entropy with Shannon entropy and Deng entropy is verified with some simple numerical examples. In the Section 4.2, the superiority of the new belief entropy compared with some other uncertainty measures is presented.

### 4.1 Compatibility with Shannon entropy

**Example 4.1.** Consider a target identification problem, if the target reported by the sensor is *a* with one hundred percent belief, then the mass function can be denoted as *m*({*a*}) = 1 in the frame of discernment *X* = {*a*}.

Shannon entropy *H*, Deng entropy *E*_*d*_ and the new belief entropy *E*_*Md*_ are calculated respectively as follows:

$$\begin{array}{cc} & H\left(m\right)=-1\times \log_{2}1=0,\\ & E_{d}\left(m\right)=-1\times \log_{2}\frac{1}{2^{1}-1}=0,\\ & E_{Md}\left(m\right)=-1\times \log_{2}\left(\frac{1}{2^{1}-1}e^{\frac{1-1}{1}}\right)=0. \end{array}$$

It is obvious that the uncertain degree for a certain event is zero. So the values of Shannon entropy, Deng entropy and the new belief entropy are all zero.

**Example 4.2.** Consider the mass function *m*({*a*}) = *m*({*b*}) = *m*({*c*}) = *m*({*d*}) = *m*({*e*}) = 0.2 in the frame of discernment *X* = {*a*, *b*, *c*, *d*, *e*}.

Shannon entropy *H*, Deng entropy *E*_*d*_ and the new belief entropy *E*_*Md*_ are calculated respectively as follows:

$$\begin{array}{cc} & H\left(m\right)=\left(-0.2\times \log_{2}0.2\right)\times 5=2.3219,\\ & E_{d}\left(m\right)=\left(-0.2\times \log_{2}\frac{0.2}{2^{1}-1}\right)\times 5=2.3219,\\ & E_{Md}\left(m\right)=\left(-0.2\times \log_{2}\left(\frac{0.2}{2^{1}-1}e^{\frac{1-1}{5}}\right)\right)\times 5=2.3219. \end{array}$$

According to Example 4.1 and 4.2, if the mass value is only assigned on the single element, the result of the new belief entropy is consistent with Shannon entropy and Deng entropy. The compatibility of the new belief entropy with Shannon entropy and Deng entropy verifies the effectiveness and rationality of the proposed belief entropy.

### 4.2 Superiority of the new belief entropy

In this section, the numerical examples are no longer appropriate for Shannon entropy, so the comparison is between the proposed belief entropy and some other uncertainty measures in Dempster-Shafer framework.

**Example 4.3.** Consider the mass function *m*({*a*, *b*, *c*, *d*, *e*}) = 1 in the frame of discernment *X* = {*a*, *b*, *c*, *d*, *e*}.

Deng entropy *E*_*d*_ and the new belief entropy *E*_*Md*_ are calculated as follows:

$$\begin{array}{cc} & E_{d}\left(m\right)=-1\times \log_{2}\frac{1}{2^{5}-1}=4.9542,\\ & E_{Md}\left(m\right)=-1\times \log_{2}\left(\frac{1}{2^{5}-1}e^{\frac{5-1}{5}}\right)=3.8000. \end{array}$$

The result shows that both Deng entropy and the new belief entropy of this vacuous mass function are bigger than that in Example 4.2. This is because the vacuous mass function in Example 4.3 means the information is totally unknown for the system, but the Bayesian mass function in Example 4.2 shows that the probability is equally distributed in the system. More information is available in Example 4.2 than the vacuous mass function in Example 4.3, so the uncertain degree in Example 4.2 should be smaller than that of the vacuous mass function. In addition, in Example 4.3, the uncertain degree indicated by the new belief entropy is smaller than that of Deng entropy, this is achieved by taking into consideration of the scale of the FOD in the BOE. That is to say, by taking into consideration of more available information, the uncertain degree measured by the new belief entropy is significantly decreased in comparison with Deng entropy. It’s also safe to say that the new belief entropy can be more accurate for uncertainty measure in this case.

In order to test the capacity and superiority of the new belief entropy, recall the example in [54] as the following example.

**Example 4.4.** Consider the mass function *m*({6}) = 0.05, *m*({3, 4, 5}) = 0.05, *m*(*T*) = 0.8 and *m*(*X*) = 0.1 in a FOD *X* = {1, 2, …, 14, 15} with fifteen elements denoted as Element 1, …, and Element 15. *T* represents a variable subset with the number of element changes from Element 1 to Element 14, as is shown in Table 2.

**Table 2:** Modified Deng entropy with a variable element in *T*.

Cases	Deng entropy	The modified Deng entropy
*T* = {1}	2.6623	2.5180
*T* = {1, 2}	3.9303	3.7090
*T* = {1, 2, 3}	4.9082	4.6100
*T* = {1, …, 4}	5.7878	5.4127
*T* = {1, …, 5}	6.6256	6.1736
*T* = {1, …, 6}	7.4441	6.9151
*T* = {1, …, 7}	8.2532	7.6473
*T* = {1, …, 8}	9.0578	8.3749
*T* = {1, …, 9}	9.8600	9.1002
*T* = {1, …, 10}	10.6612	9.8244
*T* = {1, …, 11}	11.4617	10.5480
*T* = {1, …, 12}	12.2620	11.2714
*T* = {1, …, 13}	13.0622	11.9946
*T* = {1, …, 14}	13.8622	12.7177

**Fig 1:**
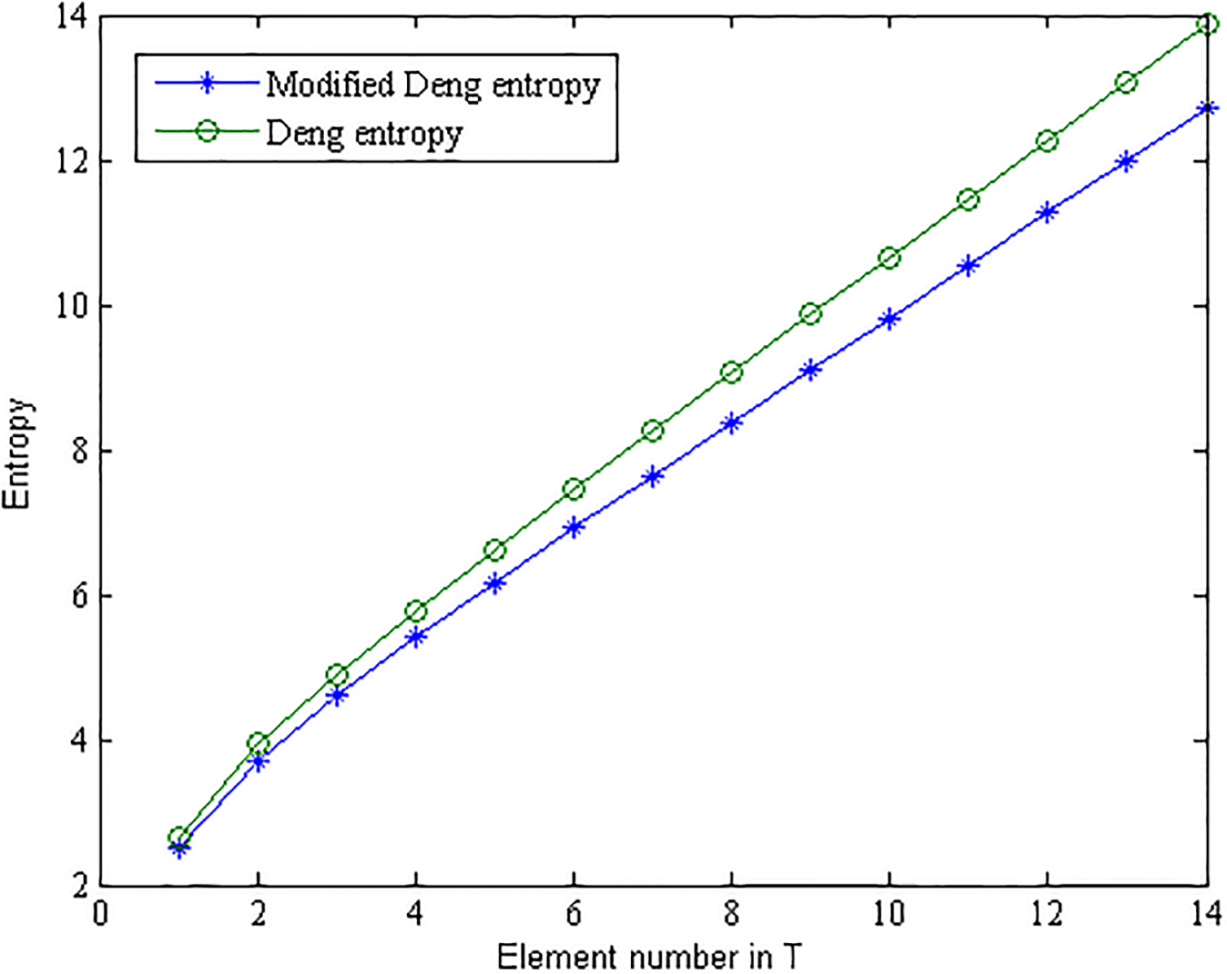
Comparison between the modified belief entropy and Deng entropy.

Deng entropy *E*_*d*_ and the modified belief entropy *E*_*Md*_ are calculated with a changed proposition *T*, the results are shown in Table 2 and Fig 1.

Table 2 and Fig 1 show that the modified belief entropy is smaller than Deng entropy. This is reasonable, because more information in the BOE is taken into consideration within the modified belief entropy. The proposed method has a less information loss than Deng entropy.

**Fig 2:**
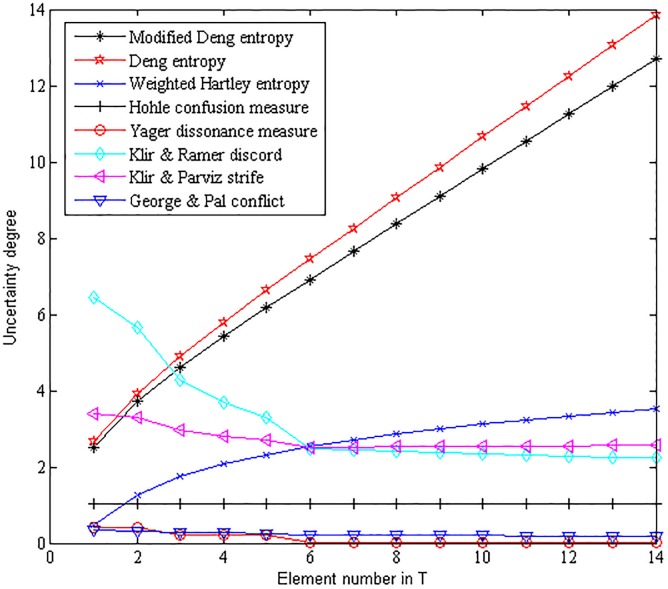
Comparison among different uncertainty measures.

Fig 2 shows the results of comparison between the modified belief entropy and some other typical uncertainty measures in Dempster-Shafer framework.

In Fig 2, the uncertain degree measured by Hohle’s confusion measure never changes with the variation of the element number in proposition *T*, thus it cannot measure the variance of uncertain degree in this case. Similar to Hohle’s confusion measure, Yager’s dissonance measure has a limited capacity of uncertainty measure in this case, both of these two methods can’t measure the change in proposition *T*. The uncertain degree measured by Klir & Ramer’s discord measure, Klir & Parviz’s strife measure and George & Pal’s conflict measure is decreasing with the increasing of the element number in the proposition *T*. Thus, the confusion measure, the dissonance measure, the discord measure, the strife measure and the conflict measure can’t effectively measure the rising of the uncertain degree along with the increasing of the element number in the proposition *T*.

It seems that the uncertain degree measured by Dubois & Prade’s weighted Hartley entropy, Deng entropy and the modified belief entropy is rising significantly along with the increasing of the element number in proposition *T*. However, the weighted Hartley entropy and Deng entropy can’t distinguish the different uncertain degree among BOEs with similar BPAs on different FODs, as is shown in Example 3.1. More importantly, by taking into consideration of the scale of the FOD and the cardinality of each proposition simultaneously, the uncertain degree measured by the modified belief entropy is significantly decreased in comparison with Deng entropy. The proposed modified belief entropy takes advantage of more valuable information in BOE, which ensures it to be more reasonable and effective for uncertainty measure in Dempster–Shafer framework.

## 5 A case study

In order to show the effectiveness and the application prospect of the modified belief entropy, the case study in [69] and the fault diagnosis method in [10] are recalled in this section. While performing the fault diagnosis method in [10], this paper changes Deng entropy into the new belief entropy.

Recall the example in [69]. Three fault types are denoted as *F*_1_, *F*_2_ and *F*_3_, the fault hypothesis set is Θ = {*F*_1_, *F*_2_, *F*_3_}, three sensors report the diagnosis results independently, the diagnosis results are modelled as BOEs, denoted as *E*_1_, *E*_2_ and *E*_3_, the BPAs of the diagnosis results are shown in Table 3.

**Table 3:** BPAs of the case study [69].

Sensor report	{*F*_1_}	{*F*_2_}	{*F*_2_, *F*_3_}	Θ
*E*_1_: *m*_1_(⋅)	0.60	0.10	0.10	0.20
*E*_2_: *m*_2_(⋅)	0.05	0.80	0.05	0.10
*E*_3_: *m*_3_(⋅)	0.70	0.10	0.10	0.10

Based on the sensor reports in Table 3, which one is the fault that happens now, *F*_1_, *F*_2_ or *F*_3_? With Dempster’s rule of combination in Eq (5), the combination results of sensor reports are shown in Table 4. It’s hard to judge which fault has been occurred, because the combination results obtained by the conventional Dempster’s rule of combination are very close.

**Table 4:** Fused results with only Dempster’s rule of combination.

	{*F*_1_}	{*F*_2_}	{*F*_2_, *F*_3_}	Θ
Fused results	0.4519	0.5048	0.0336	0.0096

In order to handle this problem, in [10], a fault diagnosis method based on Deng entropy is proposed, the reliability of sensor data will be modelled as a weight of each BOE. Follow the fault diagnosis method in [10], the weight of the *i*th BOE (*i* = 1, 2, 3) is defined as the product of a static reliability *w*_*s*_(*i*) and a dynamic reliability *w*_*d*_(*i*), denoted as follows [10]:

$$\begin{array}{c} w\left(i\right)=w_{s}\left(i\right)\times w_{d}\left(i\right), \end{array}$$ (24)

where the static reliability *w*_*s*_(*i*) of each BOE is listed in Table 5, and the dynamic reliability *w*_*d*_(*i*) is defined as follows [10]:

$$\begin{array}{c} w_{d}\left(i\right)=Crd\left(i\right)\times \frac{E_{d}\left(m_{i}\right)}{\max(E_{d}\left(m_{i}\right))}, \end{array}$$ (25)

where *Crd*(*i*) is the credibility degree of the *i*th BOE *E*_1_, *E*_*d*_(*i*) is Deng entropy of the *i*th BOE *E*_1_, max(*E*_*d*_(*i*)) is the maximum of Deng entropy value among all the BOEs. The value of *Crd*(*i*) and *E*_*d*_(*i*) of each BOE in [10] is shown in Table 5.

**Table 5:** Parameter of the case study [10].

	*E*_1_	*E*_2_	*E*_3_
*w*_*s*_(*i*)	1.0000	0.2040	1.0000
*Crd*(*i*)	1.0000	0.5523	0.9660
*E*_*d*_(*i*)	2.2909	1.3819	1.7960

Based on Eqs (24) and (25), the weight of each BOE based on the new belief entropy *E*_*Md*_(*i*) is defined as follows:

$$\begin{array}{c} w^{\prime }\left(i\right)=w_{s}\left(i\right)\times Crd\left(i\right)\times \frac{E_{Md}\left(m_{i}\right)}{\max(E_{Md}\left(m_{i}\right))}. \end{array}$$ (26)

The modified belief entropy of each BOE is calculated as follows:

$$\begin{array}{ccc} E_{Md}\left(m_{1}\right) & = & -\sum_{A\subseteq X}m_{1}\left(A\right)\log_{2}\left(\frac{m_{1}\left(A\right)}{2^{\left|A\right|}-1}e^{\frac{|A|-1}{\left|X\right|}}\right)\\ & = & -0.6\log_{2}\left(\frac{0.6}{2^{1}-1}e^{\frac{1-1}{3}}\right)-0.1\log_{2}\left(\frac{0.1}{2^{1}-1}e^{\frac{1-1}{3}}\right)-0.1\log_{2}\left(\frac{0.1}{2^{2}-1}e^{\frac{2-1}{3}}\right)-0.2\log_{2}\left(\frac{0.2}{2^{3}-1}e^{\frac{3-1}{3}}\right)\\ & = & 2.0505, \end{array}$$

$$\begin{array}{ccc} E_{Md}\left(m_{2}\right) & = & -\sum_{A\subseteq X}m_{2}\left(A\right)\log_{2}\left(\frac{m_{2}\left(A\right)}{2^{\left|A\right|}-1}e^{\frac{|A|-1}{\left|X\right|}}\right)\\ & = & -0.05\log_{2}\left(\frac{0.05}{2^{1}-1}e^{\frac{1-1}{3}}\right)-0.8\log_{2}\left(\frac{0.8}{2^{1}-1}e^{\frac{1-1}{3}}\right)-0.05\log_{2}\left(\frac{0.05}{2^{2}-1}e^{\frac{2-1}{3}}\right)-0.1\log_{2}\left(\frac{0.1}{2^{3}-1}e^{\frac{3-1}{3}}\right)\\ & = & 1.2617, \end{array}$$

$$\begin{array}{ccc} E_{Md}\left(m_{3}\right) & = & -\sum_{A\subseteq X}m_{3}\left(A\right)\log_{2}\left(\frac{m_{3}\left(A\right)}{2^{\left|A\right|}-1}e^{\frac{|A|-1}{\left|X\right|}}\right)\\ & = & -0.7\log_{2}\left(\frac{0.7}{2^{1}-1}e^{\frac{1-1}{3}}\right)-01\log_{2}\left(\frac{0.1}{2^{1}-1}e^{\frac{1-1}{3}}\right)-0.1\log_{2}\left(\frac{0.1}{2^{2}-1}e^{\frac{2-1}{3}}\right)-0.1\log_{2}\left(\frac{0.1}{2^{3}-1}e^{\frac{3-1}{3}}\right)\\ & = & 1.6517. \end{array}$$

Now, it’s clear that max(*E*_*Md*_(*m*_*i*_)) = *E*_*Md*_(*m*_1_) = 2.0505. Based on Eq (26), the weight of each BOE based on the modified belief entropy is calculated as follows:

$$\begin{array}{ccc} w^{\prime }\left(1\right) & = & w_{s}\left(1\right)\times Crd\left(1\right)\times \frac{E_{Md}\left(m_{1}\right)}{E_{Md}\left(m_{1}\right)}=1\times 1\times \frac{2.0505}{2.0505}=1,\\ w^{\prime }\left(2\right) & = & w_{s}\left(2\right)\times Crd\left(2\right)\times \frac{E_{Md}\left(m_{2}\right)}{E_{Md}\left(m_{1}\right)}=0.2040\times 0.5523\times \frac{1.2617}{2.0505}=0.0693,\\ w^{\prime }\left(3\right) & = & w_{s}\left(3\right)\times Crd\left(3\right)\times \frac{E_{Md}\left(m_{3}\right)}{E_{Md}\left(m_{1}\right)}=1\times 0.9660\times \frac{1.6517}{2.0505}=0.7781. \end{array}$$

With a normalization step, the weight of each BOE is as follows:

$$\begin{array}{ccc} w^{\prime \prime }\left(1\right) & = & \frac{w^{\prime }\left(1\right)}{w^{\prime }\left(1\right)+w^{\prime }\left(2\right)+w^{\prime }\left(3\right)}=0.5143,\\ w^{\prime \prime }\left(2\right) & = & \frac{w^{\prime }\left(2\right)}{w^{\prime }\left(1\right)+w^{\prime }\left(2\right)+w^{\prime }\left(3\right)}=0.0375,\\ w^{\prime \prime }\left(3\right) & = & \frac{w^{\prime }\left(3\right)}{w^{\prime }\left(1\right)+w^{\prime }\left(2\right)+w^{\prime }\left(3\right)}=0.4212. \end{array}$$

Now, the BBAs in Table 3 can be modified with the normalized weight of each BOE, the weighted BBA of each propostion is calculated as follows:

$$\begin{array}{cc} & m\left(\left\{F_{1}\right\}\right)=w^{\prime \prime }\left(1\right)\times 0.6+w^{\prime \prime }\left(2\right)\times 0.05+w^{\prime \prime }\left(3\right)\times 0.7=0.6215,\\ & m\left(\left\{F_{2}\right\}\right)=w^{\prime \prime }\left(1\right)\times 0.1+w^{\prime \prime }\left(2\right)\times 0.8+w^{\prime \prime }\left(3\right)\times 0.1=0.1263,\\ & m\left(\left\{F_{2},F_{3}\right\}\right)=w^{\prime \prime }\left(1\right)\times 0.1+w^{\prime \prime }\left(2\right)\times 0.05+w^{\prime \prime }\left(3\right)\times 0.1=0.0981,\\ & m\left(\Theta \right)=w^{\prime \prime }\left(1\right)\times 0.2+w^{\prime \prime }\left(2\right)\times 0.1+w^{\prime \prime }\left(3\right)\times 0.1=0.1541. \end{array}$$

Recall Dempster’s rule of combination in Eq (5), since there are three independent BOEs, so each weighted BPA will be fused two times with itself, the calculation is shown as follows:

$$\begin{array}{cc} & m\left(\left\{F_{1}\right\}\right)=\left(m⊕m\right)⊕m\left(\left\{F_{1}\right\}\right)=0.8951,m\left(\left\{F_{2}\right\}\right)=\left(m⊕m\right)⊕m\left(\left\{F_{2}\right\}\right)=0.0738,\\ & m\left(\left\{F_{2},F_{3}\right\}\right)=\left(m⊕m\right)⊕m\left(\left\{F_{2},F_{3}\right\}\right)=0.0240,m\left(\left\{\Theta \right\}\right)=\left(m⊕m\right)⊕m\left(\left\{\Theta \right\}\right)=0.0071. \end{array}$$

The fused results with the proposed uncertainty measure are compared with some other methods, as is shown in Table 6. Intuitively, *F*_1_ is the fault type because both the first BOE *E*_1_ and the third BOE *E*_3_ have a big belief (no less than 60%) on *F*_1_, while the second BOE *E*_2_ may come from an abnormal sensor in comparison with the other two BOEs. The fused result of Yuan et al’s method with the new measure is compatible with Fan et al’s method and Yuan et al’s method (with Deng entropy), although these three methods can overcome the shortage of Dempster’s rule of combination and lead to the right conclusion, Yuan et al’s method with the new measure has the highest belief (89.51%) on the conclusion that *F*_1_ is the fault.

**Table 6:** Fault diagnosis result with different methods.

	{*F*_1_}	{*F*_2_}	{*F*_2_, *F*_3_}	Θ
Only Dempster’s rule of combination	0.4519	0.5048	0.0336	0.0096
Fan et al’s method [69]	0.8119	0.1096	0.0526	0.0259
Yuan et al’s method. [10]	0.8948	0.0739	0.0241	0.0072
Yuan et al’s method with the proposed measure	0.8951	0.0738	0.0240	0.0071

The case study demonstrates the effectiveness of the new belief entropy. In addition, the case study shows a promising application prospect of the new uncertainty measure.

## 6 Conclusions

In information processing, each tiny piece of information is valuable. The uncertain information should be addressed cautiously, especially when there is limited available information. In this paper, a new belief entropy based on Deng entropy is proposed. The proposed method takes full advantage of uncertain information in BOE, including the mass function, the cardinality of the proposition and the scale of FOD. By addressing more available information of BOEs, the difference of uncertain degree that can’t be addressed by some other uncertainty measures now can be distinguished successfully. Numerical examples show that the new belief entropy can quantify the uncertain degree of BOE more accurately. The case study demonstrates the effectiveness and the application prospect of the new measure. Further study of this work will be focused on the application of the proposed measure. The new belief entropy provides a promising way to measure the uncertain degree in decision making, fault diagnosis, pattern recognition, risk analysis and so on.
